# 

**Published:** 1873-12

**Authors:** 


☞ RENEW YOUR SUBSCRIPTIONS. ☜

**PARTICULAR NOTICE.**

☞ *THE JOURNAL will not be sent, after the present number, to any who have not renewed their subscriptions.*

**TERMS, ALWAYS IN ADVANCE:** One copy, per annum .... $3 00

☞ Yearly subscriptions must begin with the January number.

☞ Persons who send Subscriptions for the JOURNAL will please consider the first number received after subscription as a sufficient receipt for money sent.

☞ We cannot return rejected manuscripts.

CHICAGO:

W. B. KEEN, COOKE & CO., PUBLISHERS,

NOS. 113 AND 115 STATE STREET.

☞ **Complete Sets for the year 1872, will be furnished for $3.00.**

